# Stroke frequency, but not swimming speed, is related to body size in free-ranging seabirds, pinnipeds and cetaceans

**DOI:** 10.1098/rspb.2006.0005

**Published:** 2006-12-05

**Authors:** Katsufumi Sato, Yutaka Watanuki, Akinori Takahashi, Patrick J.O Miller, Hideji Tanaka, Ryo Kawabe, Paul J Ponganis, Yves Handrich, Tomonari Akamatsu, Yuuki Watanabe, Yoko Mitani, Daniel P Costa, Charles-André Bost, Kagari Aoki, Masao Amano, Phil Trathan, Ari Shapiro, Yasuhiko Naito

**Affiliations:** 1 International Coastal Research Centre, Ocean Research Institute, The University of Tokyo2-106-1 Akahama, Otsuchi, Iwate 028-1102, Japan; 2 Graduate School of Fisheries Sciences, Hokkaido UniversityMinato-cho 3-1-1, Hakodate 041-8611, Japan; 3 National Institute of Polar Research1-9-10 Kaga, Itabashi, Tokyo 173-8515, Japan; 4 Sea Mammal Research Unit, University of St AndrewsFife KY16 8LB, UK; 5 Institute for East China Sea Research, Nagasaki UniversityTaira-machi 1551-7, Nagasaki 851-2213, Japan; 6 Center for Marine Biotechnology and Biomedicine, Scripps Institution of Oceanography, University of CaliforniaSan Diego, La Jolla, CA 92093-0204, USA; 7 Centre d'Ecologie et Physiologie Engergétiques, CNRS23 rue Becquerel, 67087 Strasbourg Cédex, France; 8 National Research Institute of Fisheries Engineering, Fisheries Research AgencyHasaki, Kamisu, Ibaraki 314-0408, Japan; 9 Center for International Cooperation, Ocean Research Institute, The University of Tokyo1-15-1 Minamidai, Nakano, Tokyo 164-8639, Japan; 10 Tokyo Institute of Technology2-12-1 Ookayama, Meguro-ku, Tokyo 152-8550, Japan; 11 Long Marine Laboratory, Department of Ecology and Evolutionary Biology, University of CaliforniaSanta Cruz, CA 95060, USA; 12 Centre d'Etudes Biologiques de Chizé-CNRSVillier en Bois, 79360 Beauvoir/Niort, France; 13 British Antarctic Survey, Natural Environment Research CouncilHigh Cross, Madingley Road, Cambridge CB3 0ET, UK; 14 Biology Department, Woods Hole Oceanographic InstitutionWoods Hole, MA 02543, USA

**Keywords:** accelerometer, power spectral density, dive, free-ranging, scaling, optimal

## Abstract

It is obvious, at least qualitatively, that small animals move their locomotory apparatus faster than large animals: small insects move their wings invisibly fast, while large birds flap their wings slowly. However, quantitative observations have been difficult to obtain from free-ranging swimming animals. We surveyed the swimming behaviour of animals ranging from 0.5 kg seabirds to 30 000 kg sperm whales using animal-borne accelerometers. Dominant stroke cycle frequencies of swimming specialist seabirds and marine mammals were proportional to *mass*^−0.29^ (*R*^2^=0.99, *n*=17 groups), while propulsive swimming speeds of 1–2 m s^−1^ were independent of body size. This scaling relationship, obtained from breath-hold divers expected to swim optimally to conserve oxygen, does not agree with recent theoretical predictions for optimal swimming. Seabirds that use their wings for both swimming and flying stroked at a lower frequency than other swimming specialists of the same size, suggesting a morphological trade-off with wing size and stroke frequency representing a compromise. In contrast, foot-propelled diving birds such as shags had similar stroke frequencies as other swimming specialists. These results suggest that muscle characteristics may constrain swimming during cruising travel, with convergence among diving specialists in the proportions and contraction rates of propulsive muscles.

## 1. Introduction

In a Friday Evening Discourse given at the Royal Institution in 1949, A. V. Hill (1950) discussed the movement of animals in relation to their dimensions, and predicted that running or swimming speed and muscle shortening speed should be independent of body size for geometrically similar animals. If true, the time required for a given movement would increase in direct proportion to linear size of animals, therefore movement frequency would be proportional to *length*^−1^ or *mass*^−1/3^. In contrast, a recent constructal theory (Bejan & Marden 2006) of swimming concluded that to minimize work, optimal stroke frequency should scale with *mass*^−1/6^ and optimal speed with *mass*^1/6^. Empirical data on stroke frequency and swimming speed in relation to mass over a wide range of animal sizes are the key to address this discrepancy.

Direct observations have often been used to record movements of flying animals (Pennycuick 1990, 1996) and several studies have investigated the swimming behaviour of diving animals in detail at shallow depths in aquaria (Clark & Bemis 1979; Johansson & Aldrin 2002). However, captive animals have no reason to swim optimally, which encourages recording of animals' movements *in situ*. Recently, small-sized accelerometers have been developed that now facilitate a wide survey of locomotion in free-ranging aquatic animals (Johnson & Tyack 2003; Watanuki *et al*. 2003; Sato *et al*. 2004). We therefore deployed an accelerometer on each free-ranging animal for a specified period, and retrieved it when the instrumented animal was recaptured or using auto-releasing systems (Miller *et al*. 2004; Watanabe *et al*. 2004). During the deployment periods, animals swam, dove and flew under natural conditions. In most cases, they repeated breath-hold dives to capture prey at depth. Oxygen is stored in the body at the water surface and is depleted underwater through metabolic processes, including locomotion. Efficient locomotion is critical for diving foragers to prolong their dive times and increase the proportion of time at the foraging depth. Our new methods using the animal-borne accelerometers along with swim speed and depth sensors provide the required information on natural swimming behaviour of free-ranging animals in contexts where they are expected to swim efficiently.

## 2. Material and methods

We compared the stroke frequencies and swimming speeds of a range of animals in relation to their body sizes. Owing to morphological differences among species, body mass was used as an index of body size. Morphological measurements were used to estimate mass for adult Weddell seals (Sato *et al*. 2003), leatherback turtles and sperm whales (Miller *et al*. 2004). For the killer whale, we used typical sex-specific masses reported in the literature (Williams 1999; Rohr & Fish 2004). Mass of the other species was measured directly using balances. We recorded the behavioural context of each species during the period when data were collected (table 1). We specified migrating contexts, including short-distance translocations, and breath-hold diving for foraging as periods when animals are expected to swim efficiently. Seabirds were classified into one of two groups: those with a specialized swimming organ (e.g. flippers of penguins and feet of shags); and those that use the same organ for both flight and swimming (wings of auklets, guillemots and razorbills).

Field experiments using accelerometers were conducted from tropical to Antarctic regions. Detailed protocols of the field experiments were already published for the sperm whale (Amano & Yoshioka 2003; Miller *et al*. 2004), Weddell seal (Sato *et al*. 2003), Baikal seal (Watanabe *et al*. 2004), finless porpoise (Akamatsu *et al*. 2002), emperor penguin (Sato *et al*. 2005), king penguin (Sato *et al*. 2002), Adélie penguin (Sato *et al*. 2002), macaroni penguin (Sato *et al*. 2004), little penguin (Watanuki *et al*. 2006), Brünnich's guillemot (Watanuki *et al*. 2003), European shag (Watanuki *et al*. 2005), common guillemot (Watanuki *et al*. 2006), razorbill (Watanuki *et al*. 2006), rhinoceros auklet (Watanuki *et al*. 2006), chum salmon (Tanaka *et al*. 2001) and Japanese flounder (Kawabe *et al*. 2004). The location and time of the studies for other animals were as follows: killer whales (Tysfjord, Norway, November 2005; and SE Alaska, USA, July 2006); chinstrap penguins (Signy Island, South Atlantic, January 2001); gentoo penguins, black-browed albatrosses and South Georgian shags (Bird Island, South Georgia, January 2005); southern elephant seal (Kerguelen Islands, South Indian Ocean, December 2002); northern elephant seal (California, USA, March 2003); streaked shearwaters (Sangan Island, Japan, September 2004); and leatherback turtles (French Guiana, South America, May 2001–2004). Study protocols followed those of the above-mentioned published studies.

We used acceleration data loggers (D2GT and PD2GT, Little Leonardo Ltd, Tokyo; Dtag, the Woods Hole Oceanographic Institution; Johnson & Tyack 2003) to detect the stroking movement and the swim speed of animals. The D2GT was 15 mm in diameter, 53 mm in length, with a mass of 16 g in air, and recorded depth, two-dimensional acceleration and temperature. The D2GT was deployed on smaller species of penguins (macaroni and little) and flying seabirds. The Dtag (150 g in air) was used to study killer whales. Both the PD2GT and the Dtag were used for the sperm whales (two whales by PD2GT and nine whales by Dtag). The swim speed was calculated from the pitch angle from longitudinal acceleration and vertical velocity from depth data (Watanuki *et al*. 2003; Miller *et al*. 2004). There are two types of PD2GT depending on the memory size. They were used for the other animals and are 27 or 22 mm in diameter, 128 or 122 mm in length, with masses of 73 or 101 g in air, and recorded swim speed, depth, two-dimensional acceleration and temperature. The swim speed was converted from the rotation of an external propeller using a calibration line that was estimated for each animal (Sato *et al*. 2002, 2003). According to calibration experiments of the PD2GTs using a water circulation tank (Akamatsu *et al*. 2002; Kawabe *et al*. 2004) or swimming pool (Tanaka *et al*. 2001), linear relationships between rotation number of propeller and water flow speed were obtained with a high coefficient of determination larger than 0.9, which enabled us to compare swim speeds among species. The mean swim speeds were calculated during propulsive swimming. For example, the speed during the ascent phase was excluded from analyses for penguins and seabirds because they glided up to the surface using buoyant force (Sato *et al*. 2002; Watanuki *et al*. 2003). The accelerometers can measure both dynamic acceleration (such as propulsive activities) and static acceleration (such as gravity). Low-frequency components of the longitudinal acceleration, along the long axis of the body, were used to calculate the pitch angle of the animals (Sato *et al*. 2003).

We could detect the duration of each stroke cycle from the time-series data, but our goal was to determine the dominant stroke cycle frequency for each animal. The periodic properties of the acceleration signal allowed us to apply a Fourier Transform to determine the dominant frequency. Power spectral density (PSD) was calculated from the entire acceleration dataset of each animal, or a subsample during identified foraging or migration behaviour to determine the dominant stroke cycle frequency using a Fast Fourier Transformation with a computer program package, Igor Pro (WaveMetric, Inc., Lake Oswego, OR, USA). For the sperm whale, the bottom phase of the dive was not used as it is typified by body rotations, which can occur at similar rates to the fluking action.

## 3. Results

**Figure 1:**
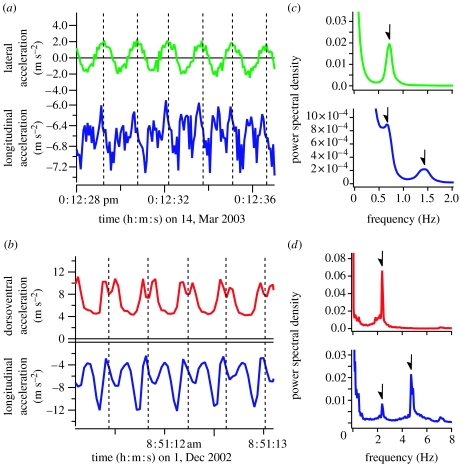
Typical examples of lateral (green line), dorsoventral (red line) and longitudinal (blue line) accelerations of (*a*) a northern elephant seal and (*b*) a macaroni penguin when they dived for foraging. The vertical broken lines delineate the separation of the stroke cycle (*a* and *b*). Power spectral density calculated from lateral (green line), dorsoventral (red line) and longitudinal (blue line) accelerations of (*c*) the northern elephant seal and (*d*) the macaroni penguin. Arrows indicate positions of peaks described in the text.

**Figure 2:**
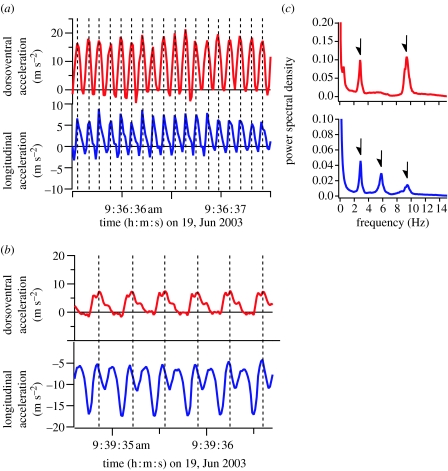
Typical examples of dorsoventral (red line) and longitudinal (blue line) accelerations of a common guillemot during (*a*) flying and (*b*) diving. (*c*) Power spectral density was calculated from dorsoventral (red line) and longitudinal (blue line) accelerations during the entire period of the data.

Seals move their rear flippers side-to-side and these movements are detected as fluctuations in lateral acceleration along the transverse axis of the body (*Mirounga angustirostris*; figure 1*a*). In penguins, a single flipper stroke cycle includes both an upstroke and a downstroke (*Eudyptes chrysolophus*; figure 1*b*). Thus, longitudinal acceleration has two positive peaks during one stroke cycle (figure 1*a*,*b*) because each flipper stroke (left-to-right or right-to-left for seals, up- or downstroke for penguins) produces thrust. The time-series data obtained from seabirds that swim and fly contain both flying and diving bouts. According to data from a common guillemot, periodical fluctuations in dorsoventral and longitudinal accelerations were synchronized during aerial flight (figure 2*a*). However, profiles during a dive were similar with those of penguins, with two positive peaks in longitudinal acceleration during a stroke cycle (figure 2*b*).

Figure 1*c*,*d* shows typical results of the PSD analyses. There is one peak in lateral acceleration and two peaks in longitudinal acceleration of a northern elephant seal (figure 1*c*). The higher frequency peak in longitudinal acceleration corresponds to each flipper stroke, while the lower frequency peak in longitudinal acceleration (and the peak in lateral acceleration) corresponds to the dominant stroke cycle frequency of this individual. In the case of a macaroni penguin, there is one peak in PSD of dorsoventral acceleration and two peaks in PSD of longitudinal acceleration (figure 1*d*). The peak in dorsoventral acceleration and the lower frequency peak in longitudinal acceleration are the dominant stroke cycle frequency of this individual. The results of PSD obtained from seabirds, which can swim and fly, were different from diving specialists. There are two peaks in PSD of dorsoventral acceleration and three peaks in PSD of longitudinal acceleration of a common guillemot (figure 2*c*). The highest frequency peaks in the PSD of dorsoventral and longitudinal accelerations corresponded to stroke cycle frequency during aerial flight, and the lowest frequency peaks correspond to stroke cycle frequency during diving.

**Figure 3:**
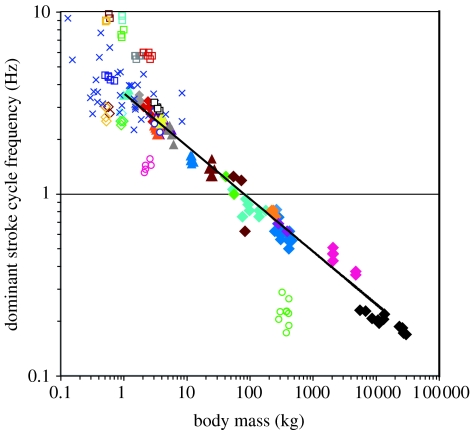
Relationship between body mass and dominant stroke cycle frequency of each individual. The symbol for each species is indicated in table 1. The line through the data points is the least squares regression for marine mammals and seabirds (closed symbols). Mean values of swimming stroke frequency and body mass for each species were used for the regression. In the case of the Weddell seal, mean values were calculated for adult females and pups, respectively. Data for flying birds (blue cross) were obtained from published studies (Pennycuick 1990, 1996).

Dominant swimming and flying stroke cycle frequencies of 25 species of animals including seals, cetaceans, penguins, seabirds, sea turtles and fishes (*n*=155 individuals) were clearly related to the body size of each individual (figure 3; table 1). Two outlier species among the swimmers included the leatherback turtle (green open circle) and Japanese flounder (pink open circle), which were not studied in a migration or a foraging context for which efficient locomotion was strongly predicted. Seabird species that use the same organ for flying and swimming (rhinoceros auklet, gold open diamond, gold open square; Razorbill, brown open diamond, brown open square; common guillemot, turquoise open diamond, turquoise open square; and Brünnich's guillemot, green open diamond, green open square) also were outlier species, both for flight and for swimming.

For the diving mammals and bird species that used specialized organs for swimming in a foraging or migration context, the dominant stroke frequency (*f*) was strongly correlated with mass (*m*). The line of best fit was obtained from a log–log analysis

$$f=3.56\;m^{-0.29},$$

with *R*^2^=0.99 (*n*=17, *p*<0.0001). The 95% confidence interval for the exponent was from −0.28 to −0.30. In contrast, the mean propulsive swim speed (*U*) among these species was independent of body mass in the log–log analysis (*U*=1.88 *m*^−0.05^, *R*^2^=0.18, *n*=17, *p*=0.09). Sperm whales of more than 30 tons, 300 kg seals and 0.5 kg seabirds all swam at mean swim speeds around 1–2 m s^−1^ during transit between the sea surface and the foraging depths (table 1).

## 4. Discussion

According to experimental measurements based on respirometers in water tunnels and the doubly labelled water technique, the optimum swim speed was proportional to *mass*^0.27^ (Videler & Nolet 1990). The constructal theoretical study (Bejan & Marden 2006) predicted that the optimal speed would be proportional to *mass*^1/6^. If the *mass*^1/6^ scaling was correct, the swim speed of a 10 000 kg sperm whale should increase by 3.68× relative to a 4 kg Adélie penguin. Instead, the mean speed of Adélie penguins was 2.0 m s^−1^ (table 1), while sperm whales in the present study swam between 1.1 and 1.9 m s^−1^, much slower than the expected value with *mass*^1/6^ scaling (2.0×3.68=7.4 m s^−1^).

Why these free-ranging animals did not follow the theoretical and experimental predictions for optimal swimming speed is a question we cannot answer now. The constructal model (Bejan & Marden 2006) did not consider that larger body size should result in a lower mass-specific maintenance cost (Williams 1999), which might support lower optimal swimming speed in larger animals. Given that speed does not vary with body size, however, the constructal model (Bejan & Marden 2006) would predict stroke frequencies to scale with *mass*^−1/3^.

There are a number of possible reasons to predict a *mass*^−1/3^ scaling of stroking frequencies in swimming specialists. Some studies have indicated that flying and swimming animals tune the Strouhal number, *St*=*fA*/*U*, which divides stroke frequency (*f*) and amplitude (*A*) by speed (*U*), within a narrow range for high propulsive efficiency (Triantafyllou *et al*. 1991; Anderson *et al*. 1998; Taylor *et al*. 2003; Rohr & Fish 2004). Since our results indicate that *U* is independent of animal size, stroke frequency *f* should be inversely proportional to stroke amplitude *A*, which is expected to be proportional to the length of animals or *mass*^1/3^ for high propulsive efficiency during foraging dives and migration.

The isometric model proposed by Hill (1950) predicted that geometrically similar animals should have running or swimming speeds independent of body size, and that stride frequency would be proportional to *mass*^−1/3^. He argued that the work that a muscle can produce depends upon its size, i.e. as the cube of the linear dimension of the animal (Hill 1950). The kinetic energy developed in a limb depends upon its mass (the cube of the linear dimension) and the square of its velocity. Thus, geometrically similar animals are predicted to move their limbs at the same speed, and consequently run or swim at the same linear speed, with stride frequencies scaling with *mass*^−1/3^.

Another explanation for a *mass*^−1/3^ scaling of stroking frequencies is possible. The force produced by muscle is proportional to the cross-sectional area of muscle and the distance of movement is proportional to the length of muscle. Consequently, as Hill (1950) described, work by muscle is expected to be proportional to the mass (*m*) of muscle (cross-sectional area multiplied by length). When the muscle is moving at a certain frequency (*f* Hz=*f* s^−1^), power produced by the muscle is expressed as *amf* (where ‘*a*’ is constant). When an animal is swimming at a certain speed (*U* m s^−1^), the mechanical power to counteract drag is 0.5*ρSC*_d_*U*^3^ (where *ρ* is the density of seawater, *S* is the referenced area of the animal and *C*_d_ is the drag coefficient). The muscle power should equal the mechanical power. Thus, we obtain the following equation

$$f=\frac{\rho SC_{\text{d}}U^{3}}{2am}.$$

According to the present study, swim speed (*U*) was independent of body size, therefore the frequency is expected to be proportional to area divided by mass (*S*/*m*), which is expected to be proportional to the *length*^−1^ or *mass*^−1/3^.

Results of the present study were obtained from morphologically diverse animals. Nonetheless, *f* is inversely proportional to *m*^−0.29^, close to the predicted value of *mass*^−1/3^, implying that diving specialists among seabirds and marine mammals have evolved similar proportions of propulsive muscles and muscle contraction rates during cruising travel.

The scaling relationship was very strong among swimming specialists during contexts when they were predicted to swim efficiently. Moreover, interesting deviations from the regression line (see figure 3) imply that morphological, ecological and physiological factors may affect the relationship between stroke frequency and body size. Birds that use their wings only for swimming would have a much smaller wing area, mainly because water density is 800 times greater than air (Alexander 2003). Penguins use their wings only for swimming and have much smaller wings than flying birds of equal mass (Hui 1988). Larger wings are essential for aerial flight. Seabirds that use their wings to power underwater diving as well as aerial flight must balance a morphological trade-off with wing area representing a compromise. This is a probable explanation for why four species (rhinoceros auklet, gold open diamond; razorbill, brown open diamond; common guillemot, blue open diamond; and Brünnich's guillemot, green open diamond) in the present study stroked at lower frequency than other swimming specialists (figure 3) because they have larger wing areas than penguins. Small wing areas in comparison with other flying specialists could explain their relatively higher stroke frequency during aerial flight (figure 3). Their muscles cannot be contracted at the same speed in both the media. Stroke frequencies of streaked shearwaters, *Calonectris leucomelas*, and black-browed albatrosses, *Diomedea melanophris*, during aerial flight (open indigo square and open black square in figure 3) are near the regression line for stroke frequencies of swimming specialists as are other flying specialists (blue cross in figure 3) obtained from field observations (Pennycuick 1990, 1996). In the case of foot-propelled diving birds such as the European shag and South Georgian shag, they stroked at the same frequency as other swimmers (filled grey diamond and filled red diamond in figure 3). They seem to have evolved an optimal-sized foot and similar stroke frequency as other swimming specialists. It looks strange that they have higher stroke frequencies for aerial flight (open grey square and open red square in figure 3). In view of biomechanics, they may need the higher stroke frequencies to lift their bodies, made heavier by additional muscles required to move their feet for swimming.

Japanese flounders *Paralichthys olivaceus* had lower stroke frequencies than other swimming specialists (open pink circle in figure 3). Fishes are not breath-hold divers, although chum salmon, *Oncorhynchus keta*, had the same stroke cycle frequency as other divers (open blue circle in figure 3). Data for salmon were obtained when they were migrating from the open sea to their home rivers (Tanaka *et al*. 2001), when efficient locomotion is predicted. Japanese flounders usually stay on the sandy seafloor and sometimes swim for foraging or migration (Kawabe *et al*. 2004). We cannot confirm whether they needed to stroke efficiently during the study period. Leatherback turtles were characterized by a much lower stroke frequency in comparison with other swimming animals (open green circle in figure 3). These data were obtained during the inter-nesting period around their nesting grounds. There is no significant evidence that they forage actively during these periods (Wallace *et al*. 2005). This may be an important difference from other breath-holding divers foraging actively under the water. It would be interesting to test whether the stroke frequency of turtles during migration or foraging follows the same mass-scaling relationship we observed in mammals and diving specialist seabirds.

## Figures and Tables

**Table 1:** Dominant stroke cycle frequency and typical swim speed of animals. (Some animals have two symbols corresponding to values for diving (open diamond) and flying (open square), respectively. The others have one symbol for diving, except streaked shearwater and black-browed albatross which has one symbol for flying. The periods during which data were obtained are abbreviated as follows: FP, foraging period; SP, swimming period; TL, translocation experiment at the beginning of moulting season; FP^I^, foraging under isolated ice hole; IP, inter-nesting period; and MP, migrating period.)

symbol in figure 3	species	*n*	mean mass (kg)	mean stroke cycle freqency (Hz)		data obtained period	mean swim speed (m s^−1^)	source of swim speed
				swim	fly			
filled dark green diamond	sperm whale *Physeter macrocephalus*	11	15 951^a^	0.20		FP	1.6	Amano & Yoshioka (2003); Miller *et al*. (2004); present study
filled pink diamond	killer whale *Orcinus orca*	9	2962^b^	0.43		FP	1.4	present study
filled blue diamond	Weddell seal *Leptonychotes weddellii* (adult)	15	330^a^	0.63		FP	1.5	Sato *et al*. (2003)
filled turquoise diamond	Weddell seal *Leptonychotes weddellii* (pup)	8	108.0	0.85		SP	0.7	present study
filled brown diamond	Baikal seal *Phoca sibirica*	3	70.1	1.02		FP	1.1	Watanabe *et al*. (2004)
filled violet diamond	northern elephant seal *Mirounga angustirostris*	2	333.5	0.66		MP, FP	1.8	present study
filled gold diamond	southern elephant seal *Mirounga leonina*	3	236.7	0.79		TL	1.3	present study
filled green diamond	finless porpoise *Neophocaena phocaenoides*	2	48.6	1.13		SP, FP	1.3	Akamatsu *et al*. (2002)
filled brown triangle	emperor penguin *Aptenodytes forsteri*	7	24.5	1.35		FP^I^	1.7	Sato *et al*. (2005)
filled blue triangle	king penguin *Aptenodytes patagonicus*	5	11.9	1.55		FP	2.1	Sato *et al*. (2002)
filled grey triangle	gentoo penguin *Pygoscelis papua*	5	5.5	2.18		FP	2.3	present study
filled violet triangle	Adélie penguin *Pygoscelis adeliae*	17	4.2	2.46		FP	2.0	Sato *et al*. (2002)
filled gold triangle	chinstrap penguin *Pygoscelis antarctica*	7	3.8	2.54		FP	2.3	present study
filled orange triangle	macaroni penguin *Eudyptes chrysolophus*	8	3.3	2.30		FP	2.0	Sato *et al*. (2004)
filled turquoise triangle	little penguin *Eudyptula minor*	5	1.1	3.60		FP	1.8	Watanuki *et al*. (2006)
open brown diamond, open brown square	razorbill *Alca torda*	3	0.57	2.92	9.33	FP	1.6	Watanuki *et al*. (2006)
open turquoise diamond, open turquoise square	common guillemot *Uria aalge*	3	0.95	2.58	9.33	FP	1.6	Watanuki *et al*. (2006)
open green diamond, open green square	Brünnich's guillemot *Uria lomvia*	3	0.95	2.46	7.59	FP	1.4	Watanuki *et al*. (2003)
open gold diamond, open gold square	rhinoceros auklet *Cerorhincha monocerata*	3	0.53	2.71	8.96	FP	1.1	Watanuki *et al*. (2006)
filled grey diamond, open grey square	European shag *Phalacrocorax aristotelis*	5	1.6	3.25	5.70	FP	1.6	Watanuki *et al*. (2005)
filled red diamond, open red square	South Georgian shag *Phalacrocorax georgianus*	6	2.4	2.92	5.83	FP	1.7	present study
open black square	black-browed albatross *Diomedea melanophris*	4	3.4		3.00	FP		present study
open indigo square	streaked shearwater *Calonectris leucomelas*	5	0.6		4.35	FP		present study
open green circle	leatherback turtle *Dermochelys coriacea*	9	363^a^	0.22		IP	0.9	present study
open blue circle	Chum salmon *Oncorhynchus keta*	2	3.4	2.31		MP	0.6	Tanaka *et al*. (2001)
open pink circle	Japanese flounder *Paralichthys olivaceus*	5	2.4	1.42		FP	0.4	Kawabe *et al*. (2004)

a Body mass of some species was calculated using morphological measurements.

b Typical reported mass for adult male and female was used.

## Supplementary Material

Flying.mov A small insects move its wings invisibly fast, while a large bird (Wandering albatross at Kerguelen Island, Indian Ocean) flaps its wings slowly.

Walking.mov Two examples of walking animals (emperor penguins at Cape Washington, Antarctica; 15-months-old boy at Iwate Prefecture, Japan). Although an accelerometer is attached on the back of the boy, it is obvious that smaller animals move their limb quickly to walk with their mothers.
